# Supplementary figures and images for: Nuclear Localization of PTTG1 Promotes Migration and Invasion of Seminoma Tumor through Activation of MMP-2
Source: Cancers (Basel). 2021 Jan 8;13(2):212. doi: 10.3390/cancers13020212 (PMC7826632; doi:10.3390/cancers13020212)

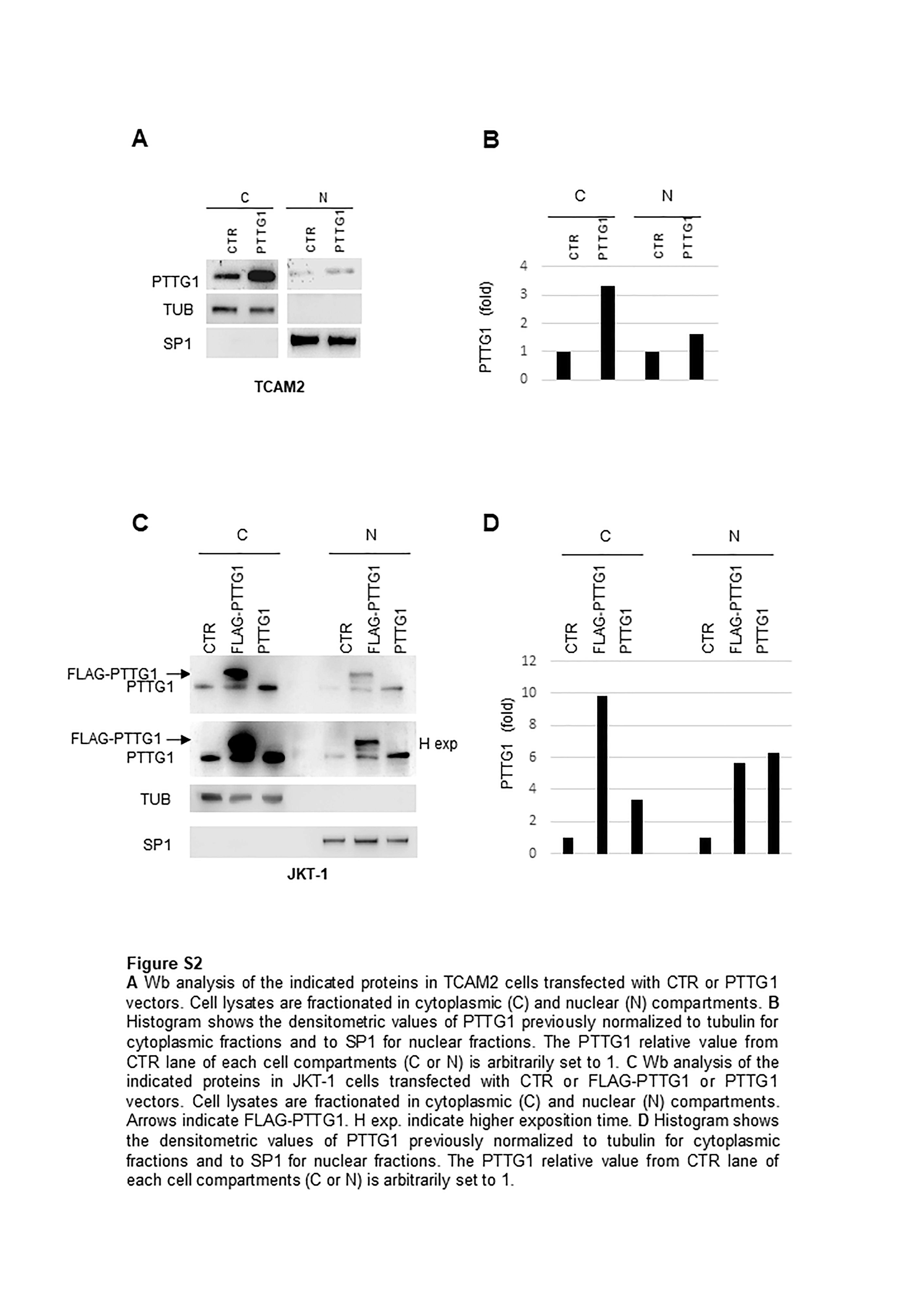

Supplement: Supplementary file 1 [file cancers-13-00212-s001.zip › Figure S2.TIF]

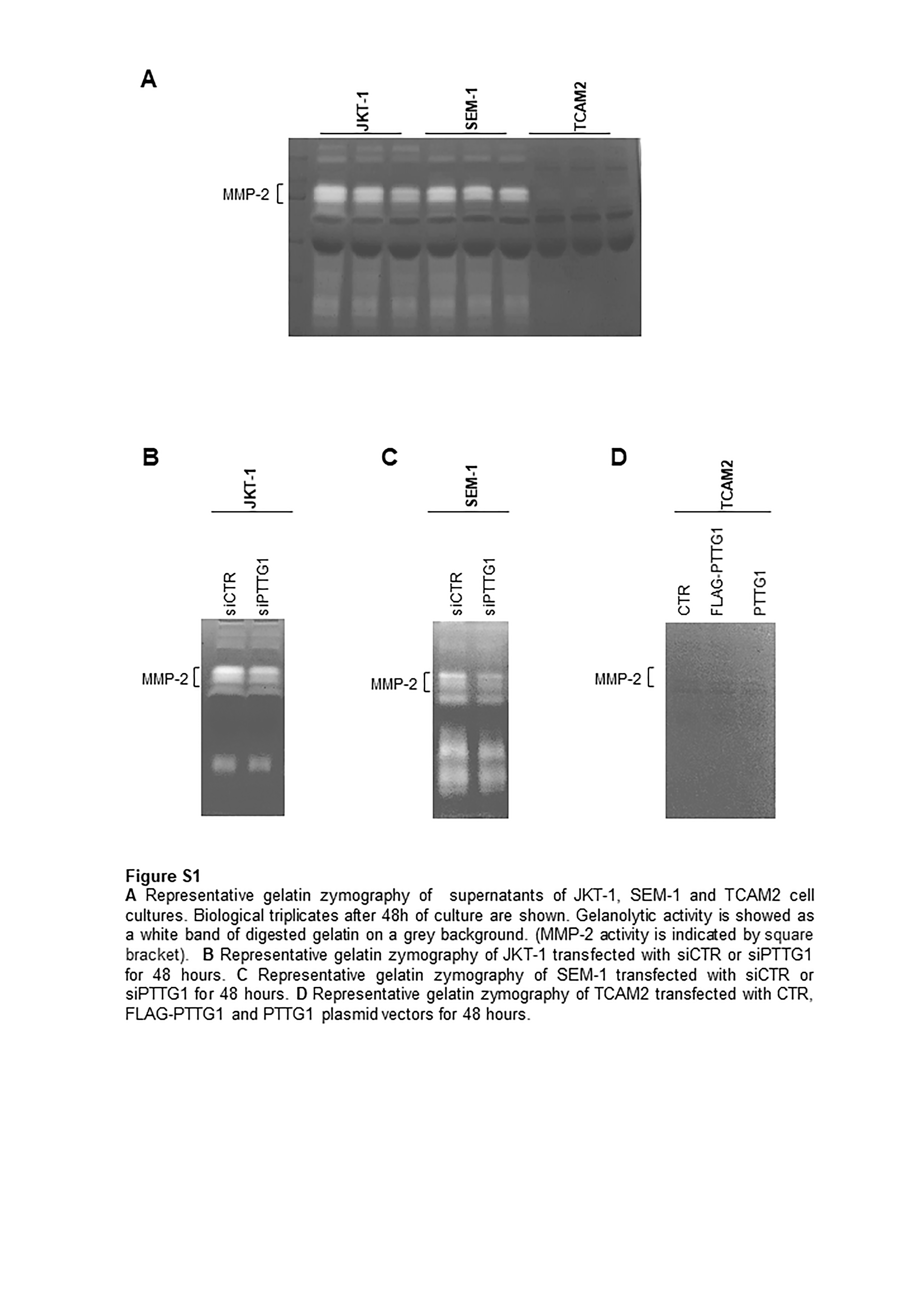

Supplement: Supplementary file 1 [file cancers-13-00212-s001.zip › Figure S1.TIF]
